# Self-Supervised Decoupled Polarization Image Dehazing with an Angle-of-Polarization Frequency-Domain Prior

**DOI:** 10.3390/jimaging12080336

**Published:** 2026-07-24

**Authors:** Lanfeng Cui, Yi Zong, Fanqiang Kong, Jianxin Li

**Affiliations:** 1 School of Astronautics, Nanjing University of Aeronautics and Astronautics, Nanjing 210016, China; 2 School of Electronic and Optical Engineering, Nanjing University of Science and Technology, Nanjing 210094, China

**Keywords:** image dehazing, polarization image, self-supervised learning, AoP frequency prior, decoupling network

## Abstract

This paper proposes a self-supervised polarization image dehazing method with an angle-of-polarization (AoP) frequency-domain prior for strong scattering dense-haze scenarios. The method formulates dehazing as the recovery of the clear object-radiance polarization field, rather than only restoring a haze-free intensity image. By analyzing real polarized hazy images, we observe that atmospheric AoP is dominated by low-frequency components, while object-radiance AoP contains richer local variations. Based on this observation, an AoP frequency-domain prior is incorporated into the polarization scattering model to guide the separation of object radiance and atmospheric polarization. A two-stage self-supervised training framework is then developed, where physical priors and the AoP prior provide stable component estimates, followed by joint optimization through scattering reconstruction consistency. In the object-radiance branch, a spatial-frequency dual-domain enhancement module is designed to capture both global haze degradation and local structural details. Experiments on a self-collected real short-wave infrared polarized hazy image dataset demonstrate that the proposed method achieves better target visibility, structural restoration, and quantitative performance than existing methods under dense-haze and strong scattering conditions.

## 1. Introduction

In natural scenes, imaging quality is significantly affected by adverse weather conditions such as fog, haze, and poor air quality. Suspended water droplets and dust particles in the atmosphere scatter and absorb light, thereby degrading image visibility, contrast, and color fidelity. This degradation severely impacts computer vision applications, including surveillance, intelligent transportation systems, and object recognition. Therefore, image dehazing plays a crucial role in improving visual quality and ensuring the reliability of such systems [1,2,3]. In recent years, image dehazing has been extensively studied, and existing methods can be broadly categorized into three groups: non-physical model-based methods, physical model-based methods, and deep learning-based methods. Traditional enhancement-based approaches (e.g., Retinex, wavelet transform, and homomorphic filtering) improve visual quality by adjusting image statistics; however, they typically ignore the underlying physical mechanisms of haze formation and thus perform poorly in complex scenarios [4,5,6].

Physical model-based methods explicitly model light propagation and atmospheric scattering. Representative approaches based on the atmospheric scattering model estimate scene radiance by recovering transmission and atmospheric light. For example, the Dark Channel Prior (DCP) exploits the observation that, in haze-free images, at least one color channel tends to have very low intensity in local patches, enabling effective estimation of transmission [7]. Subsequent works introduce regularization strategies (e.g., total variation) to refine the results. Despite their effectiveness, these methods often suffer from inaccurate transmission estimation and loss of fine details [8,9,10,11,12,13,14,15,16,17,18].

Polarization-based methods, on the other hand, exploit the polarization characteristics of light during scattering to obtain additional information, thereby improving dehazing performance [19,20]. Rowe et al. proposed a polarization-difference imaging method to enhance target contrast using orthogonal polarization images [21]. Schechner et al. established a classical polarization dehazing framework based on the atmospheric scattering model, enabling the estimation of atmospheric-light components [22]. Subsequently, polarization imaging methods based on Stokes vectors further enhanced physical modeling capabilities. Liang et al. improved dehazing accuracy by estimating atmospheric parameters through multi-angle polarization observations [23].

With the development of deep learning, data-driven dehazing methods have achieved remarkable performance. Convolutional neural networks and Transformer-based models learn end-to-end mappings from hazy to clear images [24,25]. Early works such as DehazeNet [5], MSCNN [26], and AOD-Net [27] achieved significant improvements on synthetic datasets via end-to-end training. Later, multi-branch architectures such as GFN further enhanced feature representation capabilities [22,28,29]. More recently, Transformer-based methods (e.g., Deformer) have improved dehazing performance by modeling long-range dependencies [30]. Despite their superior performance in both quantitative metrics and visual quality, deep learning methods heavily depend on training data and often lack explicit physical constraints, making them prone to overfitting and physically inconsistent results.

Deep learning-based polarization dehazing methods further integrate polarization imaging models with network optimization, enabling image restoration in real-world scenes by estimating physical quantities such as atmospheric light, transmission, or haze-free images [31,32,33]. Compared with conventional dehazing networks that rely on supervision from paired haze-free images, self-supervised polarization dehazing methods can construct input reconstruction constraints based on the atmospheric scattering model, thereby reducing their dependence on haze-free ground-truth images. Specifically, ref. [32] employs three subnetworks to estimate the clear image, atmospheric component, and transmission, whereas ref. [33] introduces Fourier-domain low-pass filtering for atmospheric-light estimation. CLIP-HNet [31] introduces cross-modal semantic guidance for remote-sensing image dehazing, but it does not exploit polarization observations or the frequency characteristics of AoP. Although Ref. [33] incorporates frequency-domain processing, neither method investigates the frequency characteristics of AoP or exploits them to facilitate the separation of atmospheric and object-radiance polarization fields. However, these methods still suffer from a pronounced ill-posedness problem: the observed hazy polarization field is jointly determined by the clear object-radiance field, the atmospheric scattering component, and the transmission, making it difficult to uniquely determine each physical component using only reconstruction loss. Although the network may achieve a small reconstruction error for the input hazy image, the predicted atmospheric component and object-radiance component may not conform to the actual polarization scattering mechanism, leading to issues such as local optima, residual haze, leakage of target structures, and physical inconsistency. This ill-posedness in component decoupling becomes even more prominent under dense-haze conditions, where the atmospheric scattering component is intensified and the structural information of the target is severely attenuated.

To address these challenges, we propose a self-supervised polarization field restoration method guided by an angle-of-polarization (AoP) frequency-domain prior. The main contributions of this work are summarized as follows. First, motivated by the distinct AoP spectral characteristics of atmospheric and object-radiance polarization fields, we introduce an AoP frequency-domain prior as a component-level physical constraint to facilitate their decoupling within the atmospheric scattering model. Specifically, atmospheric AoP is dominated by low-frequency components, whereas object-radiance AoP contains richer local variations caused by edges and textures. Second, a two-stage self-supervised optimization strategy is developed. In the first stage, polarization physical priors and the AoP frequency-domain prior are used to obtain stable initial estimates of the atmospheric component, transmission, and object-radiance field. In the second stage, these physical components are jointly refined through atmospheric scattering reconstruction consistency, while semantic regularization is employed only as an auxiliary constraint to improve visual naturalness. Third, a spatial-frequency dual-domain enhancement (SFDE) module is designed for the object-radiance restoration branch to jointly model global haze degradation and local structural details. Experiments on a real short-wave infrared polarized hazy image dataset, together with comparisons against representative traditional polarization dehazing and deep learning-based dehazing methods, demonstrate the effectiveness of the proposed method under dense-haze and low-illumination conditions.

## 2. Materials and Methods

### 2.1. Fundamentals of Polarization-Based Image Dehazing

According to the atmospheric scattering model, a hazy image can be expressed as the combination of scene radiance *J*, atmospheric light $A_{\infty }$, and transmission *t*:

(1) $$I\left(x\right)=J\left(x\right)\cdot t\left(x\right)+A_{\infty }\cdot \left(1-t\left(x\right)\right)$$

**Figure 1:**
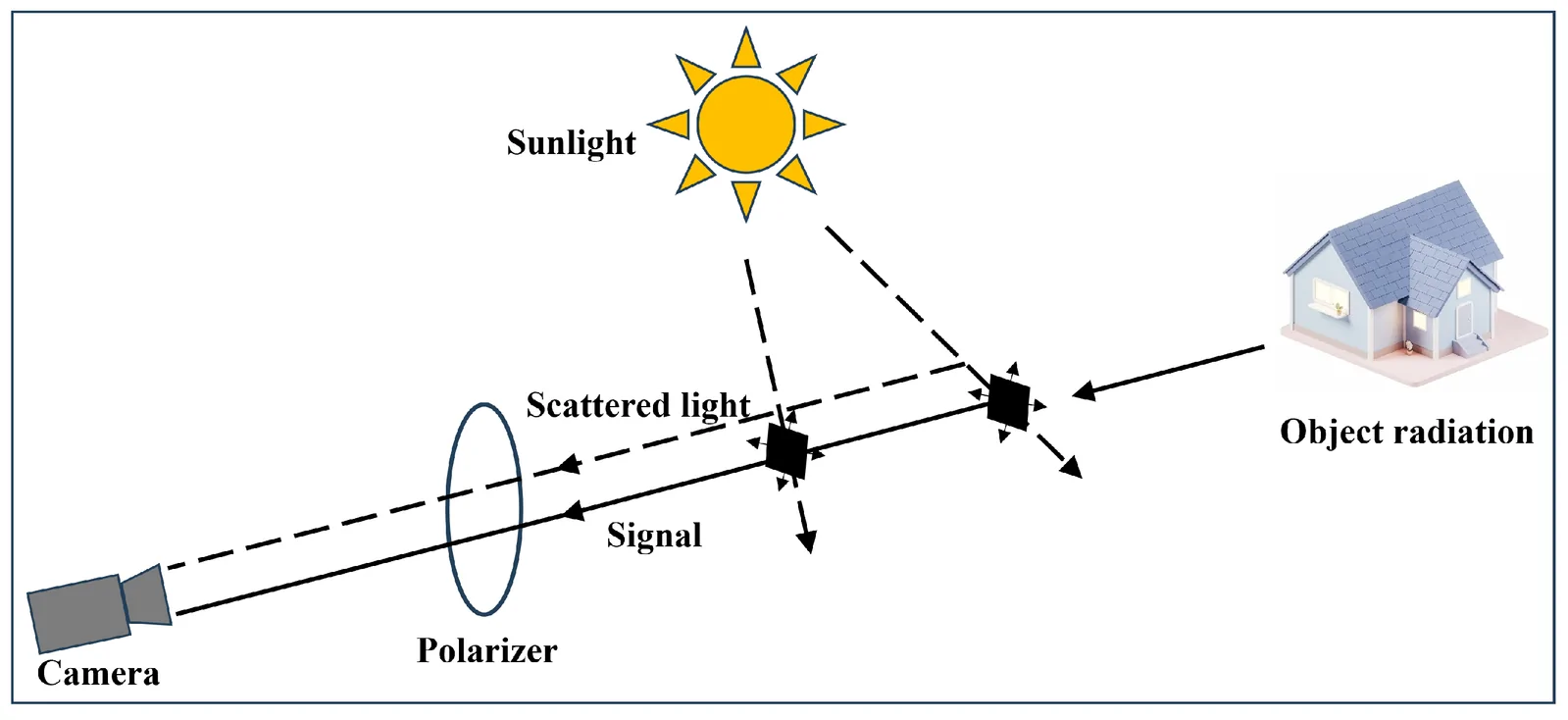
Atmospheric scattering model. Solid arrows indicate the object-radiance signal path, while dashed arrows indicate sunlight and scattered-light paths.

Constrained by Mie scattering theory, all polarization intensity components of the polarization field observed by the camera originate from the atmospheric light $A_{\infty }$, as shown in Figure 1. Leveraging this physical property, atmospheric parameters can be calculated using the atmospheric scattering model.

Given three polarization images captured at angles 0°, 60°, and 120°, denoted as $\left\{I_{0},I_{60},I_{120}\right\}$, the Stokes vector $S={\left[S_{0},S_{1},S_{2}\right]}^{T}$ is computed as

(2) $$\left[\begin{array}{ccc} S_{0} & S_{1} & S_{2} \end{array}\right]=\left[\begin{array}{ccc} \frac{2}{3}\left(I_{0}+I_{60}+I_{120}\right) & \frac{2}{3}\left(2I_{0}-I_{60}-I_{120}\right) & \frac{2}{\sqrt{3}}\left(I_{0}-I_{120}\right) \end{array}\right]$$

Based on the Stokes parameters, the AoP θ and the degree of polarization (DoP) *p* are defined as

(3) $$\theta =\frac{1}{2}\arctan\frac{S_{2}}{S_{1}}$$

(4) $$p=\frac{\sqrt{{S_{1}}^{2}+{S_{2}}^{2}}}{S_{0}}$$

The atmospheric degree of polarization $p_{A}$ is estimated by selecting the pixel with the maximum DoP. The atmospheric light $A\left(x\right)$ can then be derived as

(5) $$A\left(x\right)=\frac{\sqrt{S_{1}{\left(x\right)}^{2}+S_{2}{\left(x\right)}^{2}}}{p_{A}}$$

The global atmospheric light $A_{\infty }$ is obtained by taking the maximum value of $A\left(x\right)$. The transmission map is estimated as

(6) $$t\left(x\right)=1-\frac{A(x)}{A_{\infty }}$$

Finally, the haze-free image is recovered by

(7) $$J\left(x\right)=\frac{I\left(x\right)-A_{\infty }\left(1-t\left(x\right)\right)}{t(x)}$$

### 2.2. AoP Frequency-Domain Prior

The angle of polarization (AoP) describes the vibration direction of the linearly polarized component, reflecting the spatial directional structure of the polarized radiance field. In hazy imaging, atmospheric scattering is primarily caused by suspended particles scattering sunlight or ambient light, and its polarization direction typically varies smoothly across spatial locations, exhibiting strong spatial continuity. In contrast, the polarization angles of object radiance are influenced by surface geometry, material properties, edges, and texture details, resulting in more complex local variations. Based on this observation, we conducted a frequency-domain analysis of AoP using multiple independent full-resolution captures under clear, hazy, and sky-with-haze conditions, which were regarded as object-dominant, mixed, and atmospheric-dominant regions, respectively. To avoid correlations among adjacent image patches, the spectral statistics were calculated at the scene level. For each category, the cumulative low-frequency energy ratio was averaged across independent scenes, and the corresponding variability was also evaluated.

**Figure 2:**
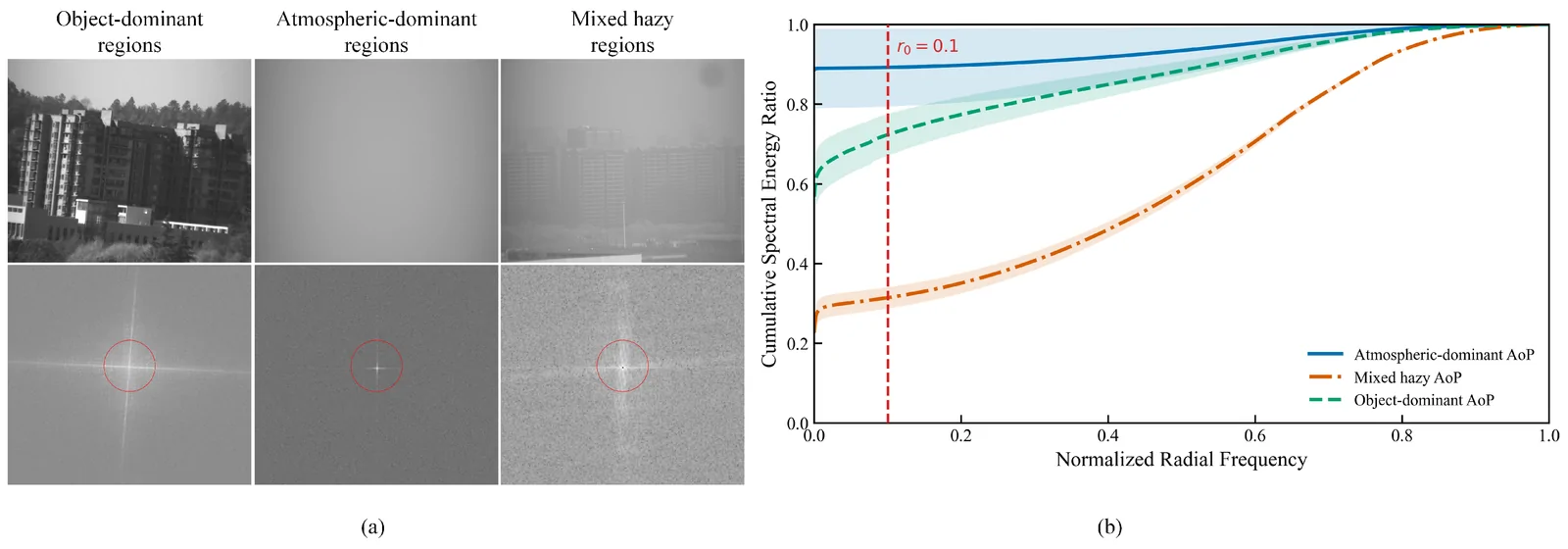
Statistical analysis of AoP frequency characteristics. (**a**) Representative images (**top**) and corresponding AoP spectra (**bottom**) of object-dominant clear, atmospheric-dominant sky-with-haze, and mixed hazy regions. The red circles indicate the low-frequency regions defined by $r_{0}=0.1$. (**b**) Mean cumulative spectral-energy curves calculated across independent scenes. The shaded regions indicate the inter-scene standard deviation.

As shown in Figure 2, the atmospheric-dominant AoP exhibits the strongest low-frequency concentration over the investigated normalized radial-frequency range. At $r_{0}=0.1$, the cumulative energy ratios of the atmospheric-dominant, object-dominant, and mixed hazy AoP fields are 89.23±27.98%, 72.35±20.72%, and 31.46±12.89%, respectively, where the values are reported as mean ± standard deviation. Their corresponding 95% confidence intervals are [83.68%, 94.78%], [68.24%, 76.46%], and [28.90%, 34.02%], respectively. Based on the cumulative spectral-energy distribution shown in Figure 2, $r_{0}=0.1$ was empirically selected to capture the dominant low-frequency atmospheric components while limiting the inclusion of object-related structural frequencies.

Based on statistical results, we construct an AoP frequency-domain prior loss to guide the self-supervised decoupling training, using the frequency structure of the atmospheric AoP to facilitate the separation of object radiance and atmospheric polarization. The following sections detail the mathematical modeling and frequency-domain processing of this prior. Let the reconstructed atmospheric Stokes parameters be

(8) $$S_{A}^{J}=S_{I}-tS_{J}=\left(S_{0}^{A},S_{1}^{A},S_{2}^{A}\right)$$

where $S_{I}$ denotes the observed hazy Stokes vector, $S_{J}$ is the clear object-radiance polarization field predicted by Net-J, and *t* is the transmission estimated by Net-t. The AoP frequency-domain prior is applied to $S_{A}^{J}$, thereby guiding the recovery of $S_{J}$ through the scattering relationship. Then the atmospheric AoP can be expressed as

(9) $$\theta_{A}\left(x\right)=\frac{1}{2}\arctan\left(\frac{S_{2}^{A}\left(x\right)}{S_{1}^{A}\left(x\right)}\right)$$

Furthermore, AoP is converted into a continuous directional representation:

(10) $$P_{A}\left(x,y\right)=\left[\cos\left(2\theta_{A}\left(x,y\right)\right),\sin\left(2\theta_{A}\left(x,y\right)\right)\right]$$

where (x,y) denotes the spatial position. This representation preserves the periodic consistency of polarization directions and avoids pseudo-high-frequency responses caused by numerical discontinuities in AoP. A two-dimensional Fourier transform is then applied to the two components of $P_{A}$, yielding

(11) $$F_{A}\left(u,v\right)=\left[F(\cos(2\theta_{A}\right.)),\left.F(\sin\left(2\theta_{A}\right))\right]$$

where F denotes the two-dimensional Fourier transform, and (u,v) denotes the frequency-domain coordinates. The corresponding spectral magnitude is defined as

(12) $$M_{A}\left(u,v\right)=\sqrt{{\left|F(\cos\left(2\theta_{A}\right))\right|}^{2}+{\left|F(\sin\left(2\theta_{A}\right))\right|}^{2}}$$

The normalized frequency radius is defined as

(13) $$\rho \left(u,v\right)=\sqrt{{\tilde{u}}^{2}+{\tilde{v}}^{2}}$$

where $\tilde{u}$ and $\tilde{v}$ are the normalized frequency coordinates. Given a low-frequency radius threshold $r_{0}$, the low-frequency energy ratio is defined as

(14) $$R_{low}\left(r_{0}\right)=\frac{\sum_{\rho \left(u,v\right)<r_{0}}M_{A}^{2}\left(u,v\right)}{\sum_{u,v}M_{A}^{2}\left(u,v\right)}$$

Based on the low-frequency dominance of atmospheric AoP, we construct an AoP frequency-domain constraint to suppress abnormal high-frequency responses in the atmospheric component. Specifically, for a frequency-domain position (u,v), its neighborhood is denoted as $\Omega_{u,v}$, and the local average spectral response is calculated as

(15) $${\bar{M}}_{A}\left(u,v\right)=\frac{1}{\left|\Omega_{u,v}\right|}\sum_{\left(p,q\right)\in \Omega_{u,v}}M_{A}\left(p,q\right)$$

To emphasize the suppression of abnormal responses in the mid- and high-frequency regions that violate the spatial smoothness of atmospheric polarization, a frequency weighting function is introduced as

(16) $$w\left(u,v\right)=\left\{\begin{array}{c} 0,\\ \rho (u,v), \end{array}\begin{array}{c} \rho \left(u,v\right)\leq r_{0}\\ \rho \left(u,v\right)>r_{0} \end{array}\right.$$

Finally, the AoP frequency-domain prior loss is defined as

(17) $$L_{AoP}=\frac{1}{N}\sum_{u,v}w(u,v)({\bar{M}}_{A}-M_{A}{)}^{2}$$

where *N* is the number of frequency-domain positions involved in the calculation.

The AoP frequency-domain prior loss is applied to $S_{A}^{J}$. By enforcing the low-frequency characteristic of atmospheric AoP, this loss guides Net-J through the scattering relationship and helps separate the object-radiance field from the atmospheric component. The proposed AoP frequency-domain prior is complementary to the atmospheric scattering reconstruction constraint. The reconstruction loss constrains whether the estimated physical components can reproduce the observed polarization images but does not uniquely determine their decomposition. In contrast, the AoP prior constrains the internal frequency structure of the inferred atmospheric polarization field, thereby providing additional guidance for component decoupling.

### 2.3. Two-Stage Self-Supervised Polarization Field Restoration Framework

Rather than directly recovering a single haze-free intensity image, this work formulates polarization image dehazing as the restoration of the clear object-radiance polarization field. Given hazy polarization observations $\left\{I_{0},I_{60},I_{120}\right\}$, the observed Stokes vector $S={\left[S_{0},S_{1},S_{2}\right]}^{T}$ is first computed according to Equation (2). The network then estimates the clear object-radiance field $S_{J}={\left[{S_{J}}_{0},{S_{J}}_{1},{S_{J}}_{2}\right]}^{T}$, the atmospheric polarization component $S_{A}$, and the transmission *t*, where the conventional dehazed image is obtained from the recovered intensity component ${S_{J}}_{0}$.

According to the atmospheric scattering model, the observed polarization field is expressed as

(18) $$S_{I}=tS_{J}+S_{A}$$

where $S_{A}=\left(1-t\right)S_{A\infty }$, and $S_{A\infty }$ denotes the global atmospheric-light Stokes vector. Since $S_{J}$, $S_{A}$, and *t* are all unknown, using only reconstruction loss for self-supervised training leads to an ill-posed decomposition problem. Therefore, a two-stage training strategy is adopted, consisting of physically guided initialization and subsequent joint optimization.

**Figure 3:**
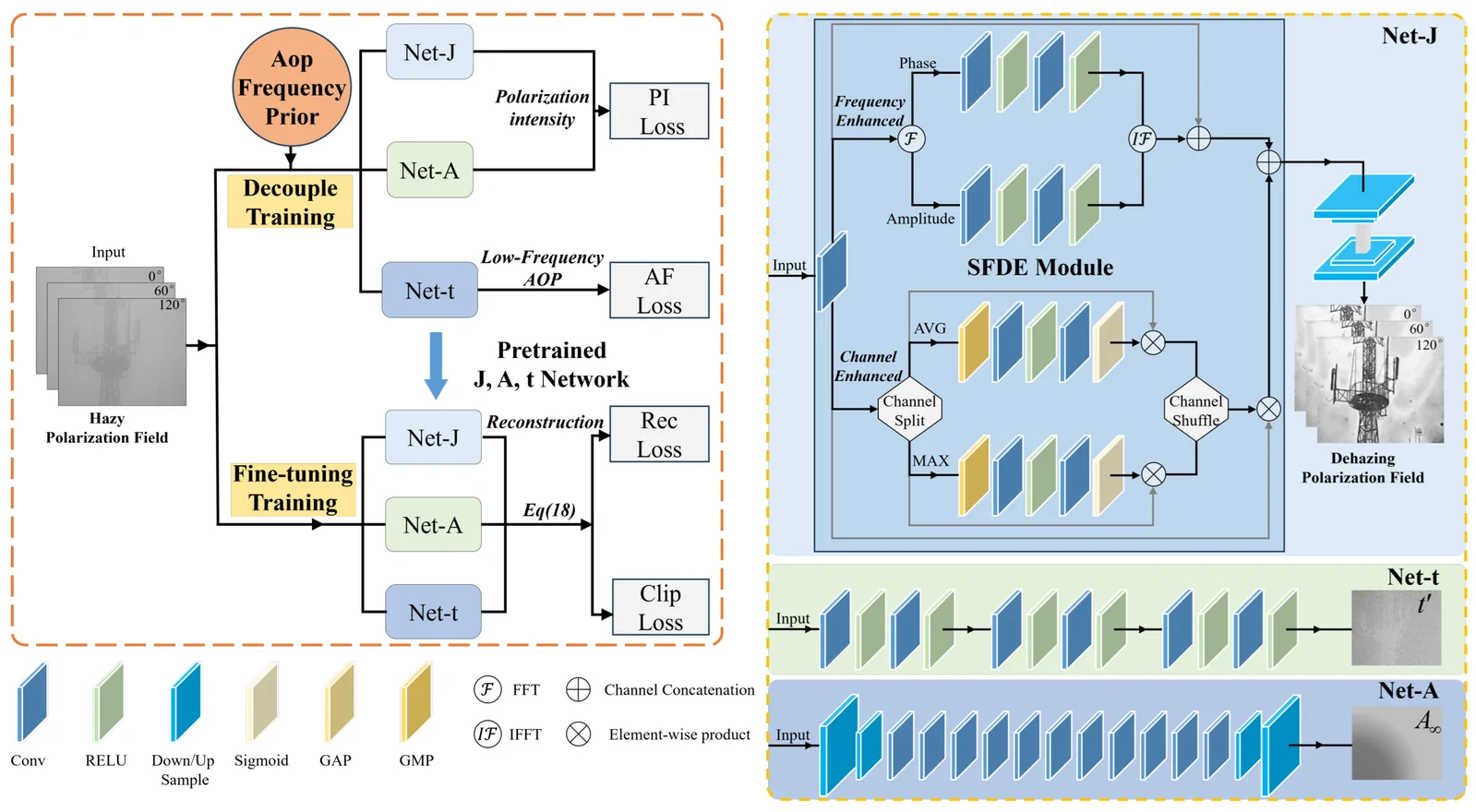
Overall architecture of the proposed two-stage framework for polarization-based image dehazing.

As shown in Figure 3, the proposed framework contains three subnetworks—Net-J, Net-A, and Net-t—which estimate the clear object-radiance polarization field $S_{J}$, the atmospheric polarization component $S_{A}$, and the transmission *t*, respectively. Net-J predicts the full Stokes representation of the clear scene, while the dehazed intensity image is obtained from ${S_{J}}_{0}$.

(19) $$L_{prior}=||Net_{A}\left(I\right)-A_{prior}{\left|\right|}_{1}+||Net_{t}\left(I\right)-t_{prior}{\left|\right|}_{1}$$

This loss leverages the physically derived priors from Section 2.1 to guide the early-stage estimation of the atmospheric component and transmission.

The object-radiance polarization field predicted by Net-J can be mapped to a corresponding atmospheric polarization field through the atmospheric scattering model. The AoP prior loss $L_{AoP}$ is then applied to this inferred atmospheric component, penalizing abnormal high-frequency responses and enforcing the expected low-frequency structure. In this way, this constraint guides Net-J to produce an object-radiance field that is more consistent with the atmospheric scattering model. The first-stage loss is formulated as

(20) $$L_{stage1}=\lambda_{Aop}L_{AoP}+\lambda_{prior}L_{prior}$$

where $\lambda_{prior}$ and $\lambda_{AoP}$ are the weights of the prior constraint and the AoP frequency-domain constraint, respectively. Through the first-stage training, the network obtains stable initial estimates of atmospheric light, transmission, and the clear polarization field, providing a physically reasonable starting point for subsequent joint optimization.

In the second stage, a reconstruction consistency constraint based on the atmospheric scattering model is introduced to jointly optimize the subnetworks. Specifically, the predictions $S_{J}$, $S_{A}$, and *t* from Net-J, Net-A, and Net-t are substituted into Equation (18) to obtain the reconstructed hazy Stokes vector:

(21) $${\hat{S}}_{I}=tS_{J}+S_{A}$$

Then, according to the linear polarization imaging relationship, the reconstructed Stokes vector ${\hat{S}}_{I}={\left[{\hat{S}}_{I0},{\hat{S}}_{I1},{\hat{S}}_{I2}\right]}^{T}$ is converted back into reconstructed images at different polarization angles:

(22) $${\hat{I}}_{a}=\frac{1}{2}\left({\hat{S}}_{I0}+{\hat{S}}_{I1}\cos2a+{\hat{S}}_{I2}\sin2a\right),a\in \left\{0^{\circ },60^{\circ },120^{\circ }\right\}$$

By enforcing consistency between the reconstructed polarization images and the input hazy polarization images, the predicted object-radiance polarization field, atmospheric polarization component, and transmission are constrained to satisfy the forward imaging process. The reconstruction consistency loss is defined as

(23) $$L_{rec}=\sum_{a\in \left\{0^{\circ },60^{\circ },120^{\circ }\right\}}{∥{\hat{I}}_{a}-I_{a}∥}_{1}$$

This loss establishes a closed-loop constraint for self-supervised training, allowing the network to be optimized without paired haze-free ground-truth images. Unlike reconstruction performed only at the intensity-image level, the proposed method imposes consistency constraints on multi-angle polarization observations, thereby preserving the physical consistency between the restored result and the original polarization measurements.

In addition, a CLIP-based semantic regularization term is introduced to improve the visual naturalness of the restored results. Specifically, real hazy images and clear images are selected as reference samples, and the frozen CLIP image encoder and text encoder are used to construct a semantic discrimination constraint between the hazy and clear domains. For the clear intensity component $S_{J0}$ output by Net-J, the CLIP semantic loss encourages semantic consistency with the clear-image domain, thereby suppressing over-enhancement, brightness imbalance, and unnatural texture artifacts. This loss is expressed as

(24) $$L_{clip}=1-\frac{\phi_{I}\left(S_{J0}\right)\cdot \phi_{T}\left(T_{C}\right)}{{∥\phi_{I}\left(S_{J0}\right)∥}_{1}{∥\phi_{T}\left(T_{C}\right)∥}_{1}}$$

where $\phi_{I}\left(\cdot \right)$ and $\phi_{T}\left(\cdot \right)$ denote the CLIP image encoder and text encoder, respectively, and $T_{C}$ represents the text prompt corresponding to the clear-image domain. This semantic constraint does not replace the physical model; instead, it serves as an auxiliary regularization term to improve the perceptual quality of the restored results. The joint optimization loss in the second stage is defined as

(25) $$L_{stage2}=\lambda_{rec}L_{rec}+\lambda_{clip}L_{clip}$$

where $\lambda_{rec}$ and $\lambda_{clip}$ are the weights of the reconstruction consistency loss and the CLIP semantic loss, respectively.

Overall, $L_{stage1}$ provides physically guided initial estimates of the atmospheric component, transmission, and clear polarization field. Then, $L_{stage2}$ refines these estimates under the atmospheric scattering model, improving both physical consistency and visual naturalness. The final dehazed image is obtained from the recovered clear Stokes intensity component ${S_{J}}_{0}$.

### 2.4. Network Architecture with the SFDE Module

Haze degradation affects both the global energy distribution and local structural details of polarization images. To enhance the representation capability of the object-radiance restoration branch, we introduce a spatial-frequency dual-domain enhancement (SFDE) module into the feature extraction stage of Net-J, as shown in Figure 3. The SFDE module consists of a frequency-enhancement branch and a channel-enhancement branch, which are designed to model global spectral degradation characteristics and inter-channel polarization response differences, respectively. The enhanced features are then fed into an encoder–decoder structure to progressively recover the clear polarization field. Let the input feature be denoted as *Z*. The frequency-enhancement branch first maps *Z* into the frequency domain using the fast Fourier transform:

(26) $$F\left(Z\right)=Z_{a}e^{jZ_{p}}$$

where $Z_{a}$ and $Z_{p}$ denote the amplitude spectrum and phase spectrum, respectively. The amplitude spectrum mainly characterizes the energy distribution of features and is closely related to the global brightness attenuation and low-frequency degradation caused by haze. In contrast, the phase spectrum preserves structural and positional information, which is important for recovering edges, textures, and object contours. Considering their different physical meanings, different convolution operations are applied to modulate the amplitude and phase spectra:

(27) $${\tilde{Z}}_{a}=f_{1\times 1}\left(Z_{a}\right),{\tilde{Z}}_{p}=f_{3\times 3}\left(Z_{p}\right)$$

where $f_{1\times 1}\left(\cdot \right)$ denotes a 1×1 convolution used to adjust the global energy response in the amplitude spectrum, and $f_{3\times 3}\left(\cdot \right)$ denotes a 3×3 convolution used to enhance local structural information in the phase spectrum. The modulated amplitude and phase spectra are then recombined and transformed back to the spatial domain through the inverse fast Fourier transform:

(28) $$Z_{FE}=F^{-1}\left({\tilde{Z}}_{a}e^{j{\tilde{Z}}_{p}}\right)$$

where $Z_{FE}$ represents the frequency-enhanced spatial feature. Through this branch, the network can adjust the haze-induced global energy degradation in the frequency domain while preserving phase information associated with target structures. On the other hand, since the input polarization images contain observations captured at different polarization angles, different feature channels exhibit distinct polarization responses. To enhance the modeling of inter-channel correlations, a channel enhancement branch is introduced into the SFDE module. This branch extracts channel-wise statistics using both global average pooling and global max pooling, and then generates channel attention weights through a shared multilayer perceptron:

(29) $$W_{c}=\sigma \left(MLP(GAP(Z))+MLP(GMP(Z))\right)$$

where GAP(·) and GMP(·) denote global average pooling and global max pooling, respectively; MLP(·) denotes the shared multilayer perceptron; and σ(·) denotes the sigmoid activation function. The channel-enhanced feature is formulated as

(30) $$Z_{CE}=W_{c}⊙Z$$

where ⊙ denotes element-wise multiplication. This branch adaptively recalibrates feature channels according to their importance for polarization field restoration, thereby enhancing informative polarization features and suppressing redundant responses. Finally, the SFDE module fuses the frequency-enhanced feature and the channel-enhanced feature as

(31) $$Z_{SFDE}=Z_{FE}+Z_{CE}$$

The fused feature contains both global degradation information in the frequency domain and polarization response information along the channel dimension, which helps Net-J restore a more stable clear polarization field under dense-haze, long-range, and low-contrast conditions. The feature $Z_{SFDE}$ is subsequently fed into an encoder–decoder structure. The encoder extracts multi-scale semantic features through stacked convolutional layers, while the decoder gradually restores the spatial resolution through upsampling. Skip connections or dense connections are further used to preserve low-level details. Finally, the output of Net-J is normalized by a sigmoid function to obtain the clear object-radiance polarization field $S_{J}$.

Net-A and Net-t are used to estimate the atmospheric polarization component and the transmission, respectively. Since the atmospheric scattering component usually exhibits strong spatial smoothness and global consistency, Net-A adopts an encoder–decoder structure to enlarge the receptive field and extract global features related to haze density, imaging distance, and illumination conditions. Its output is expressed as

(32) $$S_{A}=G_{A}\left(S_{I};\Theta_{A}\right)$$

where $G_{A}$ denotes the mapping function of Net-A, and $\Theta_{A}$ represents the corresponding network parameters.

Net-t is designed to estimate the transmission *t*. Transmission is closely related to scene depth and haze concentration and usually changes significantly near depth discontinuities and object boundaries. To reduce edge blurring caused by excessive downsampling, Net-t adopts a relatively shallow convolutional structure and preserves spatial resolution as much as possible, so as to retain local details in the transmission map. Its output is formulated as

(33) $$t=G_{t}\left(S_{I};\Theta_{t}\right)$$

where $G_{t}$ denotes the mapping function of Net-t, and $\Theta_{t}$ represents the corresponding network parameters.

## 3. Experimental Section

### 3.1. Experimental Setup and Implementation Details

**Figure 4:**
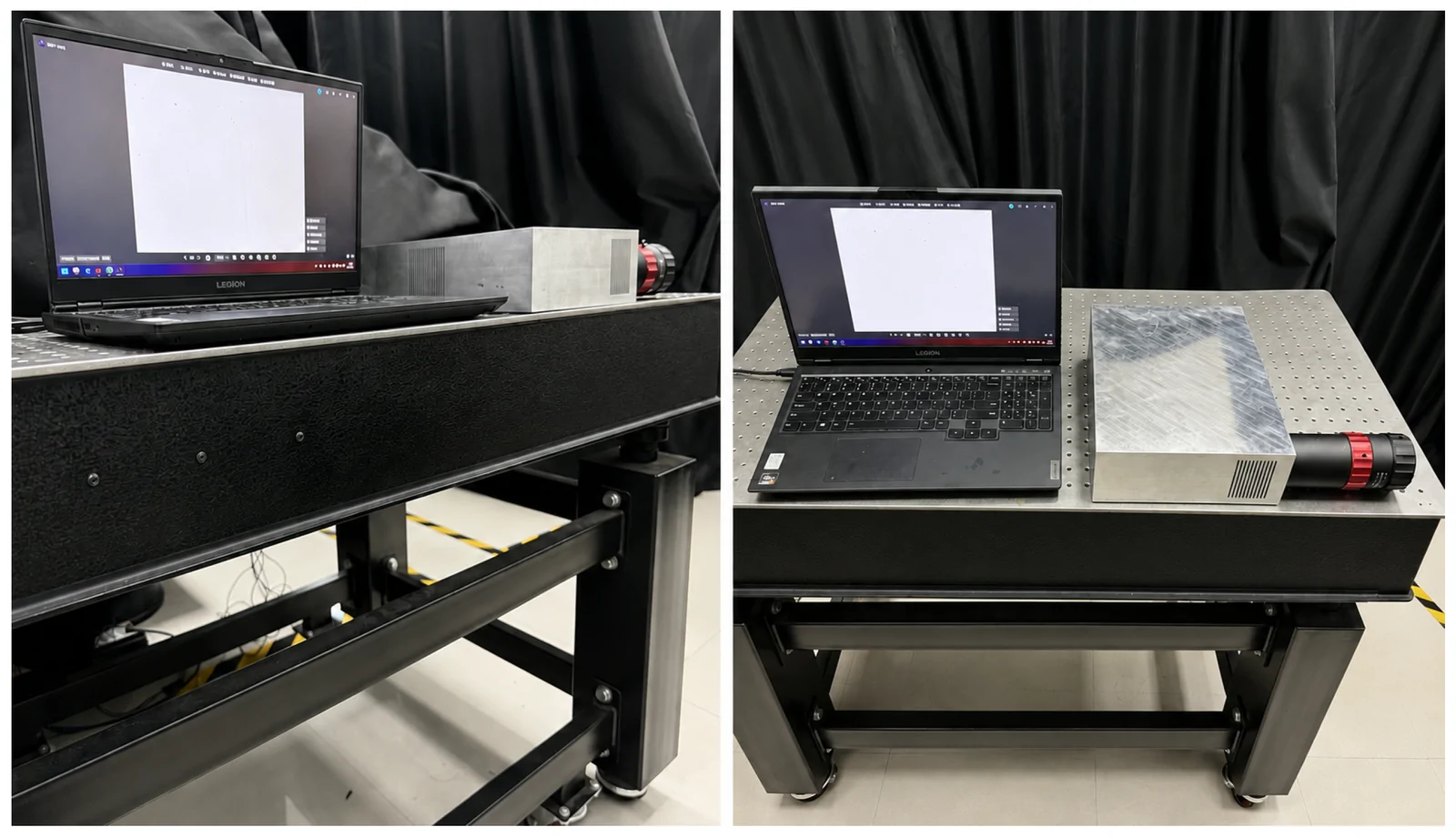
Atmospheric shortwave infrared polarization information observation system.

Most existing hazy image datasets are synthesized using atmospheric scattering models or collected under artificially generated fog, which may not fully reproduce the spatially nonuniform distribution and polarization characteristics of natural haze. Moreover, publicly available real-world polarization dehazing datasets, particularly in the short-wave infrared band, remain very limited. To obtain polarization observations under realistic atmospheric conditions, we have conducted long-term data acquisition during naturally occurring foggy weather since 2024 using a self-developed fixed short-wave infrared (SWIR, 1.2–1.7μm) polarization imaging system developed at Nanjing University of Aeronautics and Astronautics (Nanjing, China), as shown in Figure 4. The collected data cover multiple outdoor scenes with different haze densities, imaging distances, viewpoints, target scales, and background structures, including buildings, roads, vegetation, signal towers, distant mountain-view targets, and railway environments.

The system was calibrated with an integrating sphere to ensure the accuracy of both intensity and polarization measurements. Using this system, polarization images at 0°, 60°, and 120° can be simultaneously captured with a spatial resolution of 2592×2056 pixels. The full-resolution observations were first divided at the scene level to avoid content overlap between the training and test sets. A non-overlapping sliding-window strategy was applied to the training scenes to generate 30,000 groups of 256×256 polarization image patches, while 15 groups of full-resolution polarization images from held-out scenes were used for testing.

The hyperparameters were set as follows: $\lambda_{prior}=1$, $\lambda_{AoP}=1$, $\lambda_{rec}=1$, and $\lambda_{clip}=0.5$. The normalized frequency radius of the AoP frequency-domain prior was set to 0.1. The proposed network was implemented in PyTorch version 2.2.0 and trained on a computer equipped with an NVIDIA RTX 3090 GPU (NVIDIA Corporation, Santa Clara, CA, USA). The Adam optimizer was adopted with an initial learning rate of 0.0001 and a batch size of 4. The learning rate was adjusted using a cosine annealing schedule throughout training. Stage 1 and Stage 2 were trained for 15 and 15 epochs, respectively. No additional data augmentation was employed during training or testing. All convolutional layers were initialized using PyTorch default initialization.

### 3.2. Qualitative Experiments

**Figure 5:**
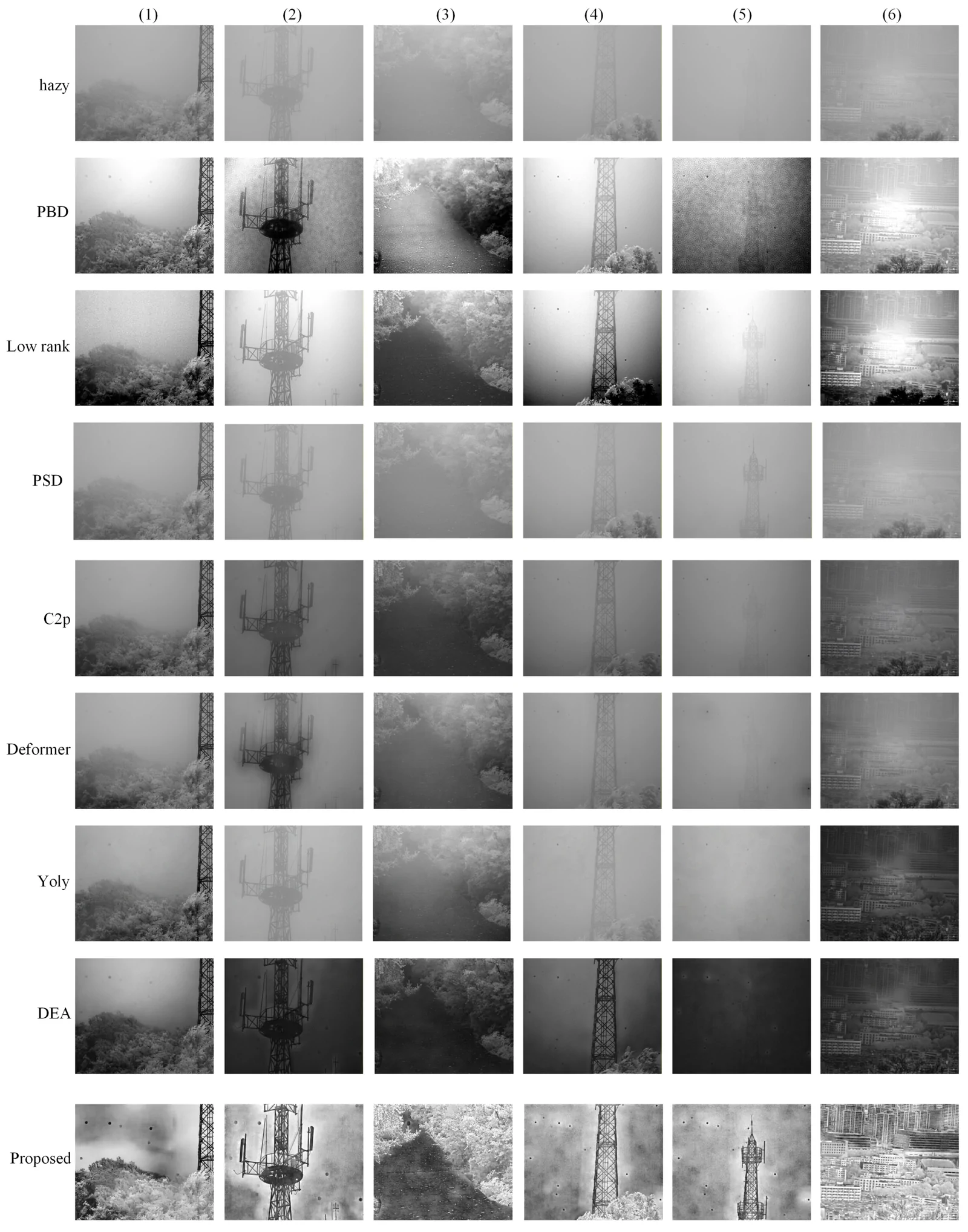
Qualitative comparison of dehazing results under light- and moderate-haze conditions. Columns (1)–(6) denote six representative test scenes.

**Figure 6:**
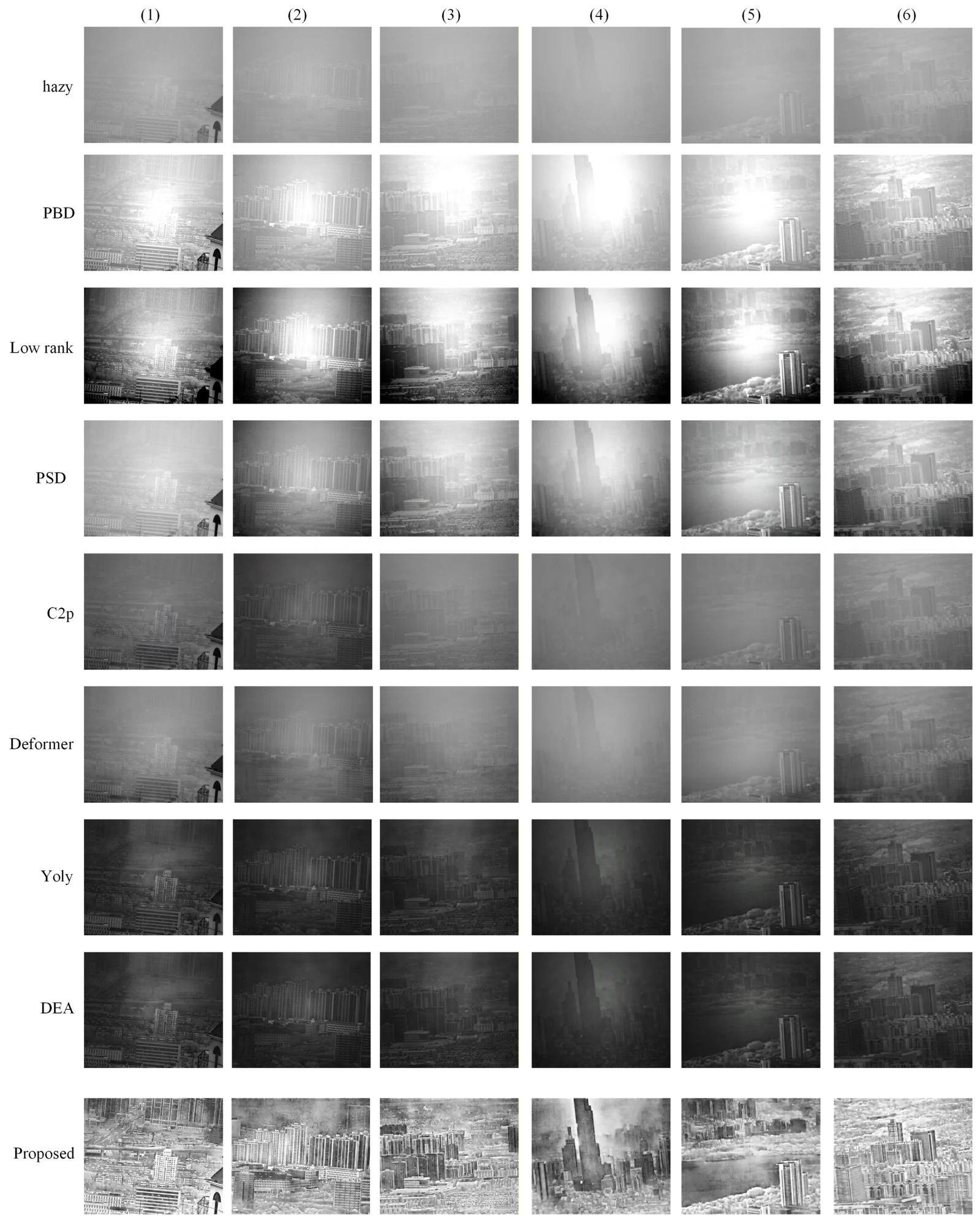
Qualitative comparison of dehazing results under dense-haze conditions. Columns (1)–(6) denote six representative test scenes.

To qualitatively evaluate the proposed method under different haze densities, we compared it with several representative dehazing methods. The comparison methods include three polarization-based dehazing approaches, namely PBD, low-rank, and PSD, together with four learning-based image dehazing methods, including C2PNet, YOLY, DEA-Net, and DeFormer. According to haze density and scene degradation severity, the test samples were divided into two groups. The first group contains light- and moderate-haze scenes, which are used to evaluate detail recovery and brightness preservation under general haze degradation. The second group contains dense-haze scenes, which are used to examine the robustness and restoration capability of different methods when target visibility is low and structural information is severely attenuated. The results of the two groups are shown in Figure 5 and Figure 6, respectively.

Figure 5 presents the dehazing results under light- and moderate-haze conditions. In these scenes, the target structures remain partially visible, but the overall image contrast is reduced, and local regions suffer from haze occlusion and detail blurring. Traditional polarization-based methods can improve image visibility to some extent; however, their performance is sensitive to the estimation of atmospheric degree of polarization and transmission, and residual haze or uneven brightness still appears in some regions. Conventional deep learning-based methods can recover certain image details in light- and moderate-haze scenes, but their results differ in terms of brightness naturalness, local texture preservation, and structural continuity. Some results tend to suffer from over-enhancement, underexposure, or unstable local details. In contrast, the proposed method improves target visibility more consistently while preserving structural information in roads, building edges, and high-intensity targets. This demonstrates that the AoP frequency-domain prior and the two-stage training strategy provide effective physical constraints and visual restoration capability under general haze degradation.

Figure 6 shows the dehazing results under dense-haze conditions. Compared with light- and moderate-haze scenes, dense-haze scenes contain a stronger atmospheric scattering component, and the target radiance is severely attenuated. As a result, many distant or weak-reflection structures are heavily obscured by haze, making object contours and local details difficult to distinguish in the original images. Therefore, this group of experiments better reflects the stability of different methods under strong scattering conditions. It can be observed that conventional deep learning-based methods exhibit a noticeable performance degradation in dense haze, often producing residual haze, insufficient brightness, structural discontinuities, or incomplete recovery of distant targets. These observations suggest that purely data-driven methods may be more sensitive to domain shifts and variations in haze density in real-world dense-haze scenes.

Traditional polarization dehazing methods, including PBD and low rank, generally perform better than most conventional deep learning-based methods in dense-haze scenes, demonstrating the importance of polarization of physical information and atmospheric scattering constraints for dehazing under strong scattering conditions. However, these methods still rely heavily on the accurate estimation of polarization parameters and atmospheric light and may suffer from local residual haze, structural blurring, or insufficient detail recovery in dense-haze regions. In comparison, the proposed method more effectively suppresses atmospheric scattering interference and recovers weak structural information, such as building contours, tower crane structures, and road edges. This advantage mainly comes from two aspects. First, the AoP frequency-domain prior directly constrains the atmospheric polarization component and reduces the leakage of target edges and texture structures into the atmospheric component. Second, the two-stage self-supervised training strategy improves the estimation stability among the clear object-radiance polarization field, the atmospheric polarization component, and the transmission. Overall, the proposed method achieves better target visibility, structural restoration, and physical consistency in dense-haze scenes, validating its effectiveness for polarization dehazing under strong scattering conditions.

**Figure 7:**
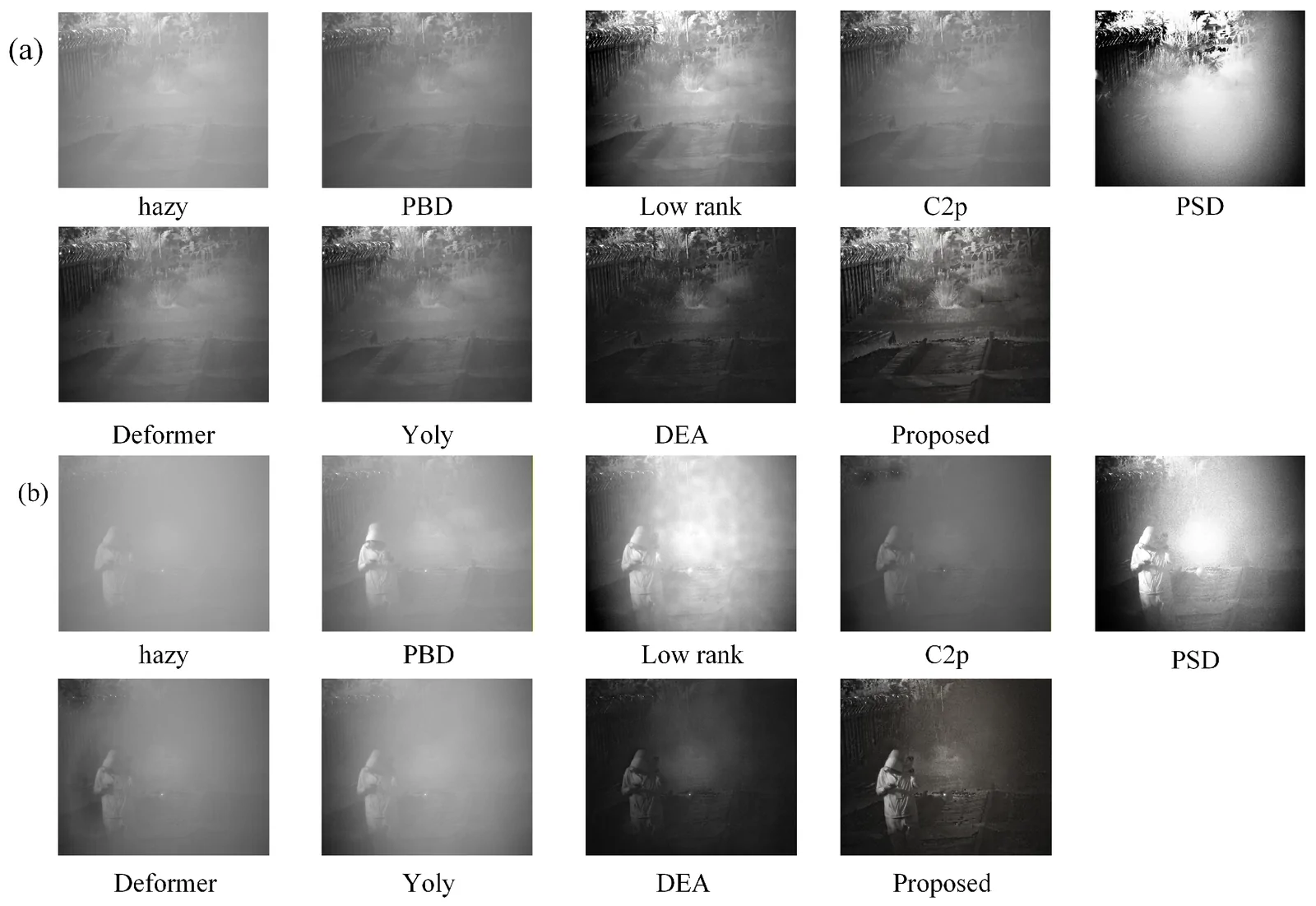
Qualitative comparison of dehazing results under dense-haze conditions in railway scenes. (**a**) Railway scene 1. (**b**) Railway scene 2.

To evaluate the robustness of the proposed method under different scene structures, two railway scenes acquired under natural dense haze were additionally selected for qualitative comparison. These scenes differ substantially from the training scenes in spatial layout, target structure, and background content. As shown in Figure 7, the proposed method effectively improves the visibility of the rails and surrounding structures while maintaining relatively continuous edges and suppressing residual haze. The results further indicate that the proposed method remains applicable to previously unseen dense-haze scenes with different structural characteristics.

To further analyze the ability of different methods to recover structural information, the Prewitt operator was used to extract edge maps from the dehazed results. Edge detection serves as an auxiliary tool for evaluating structural visibility. In general, haze reduces image contrast and weakens or even removes target edges. If a dehazing result can recover more continuous edge contours, it usually indicates better preservation of target structures. It should be noted that edge maps alone cannot serve as a complete image quality assessment criterion, since over-enhancement or noise amplification may also produce false edges. Therefore, edge maps are used here only as a structural auxiliary analysis in addition to qualitative visual comparison.

**Figure 8:**
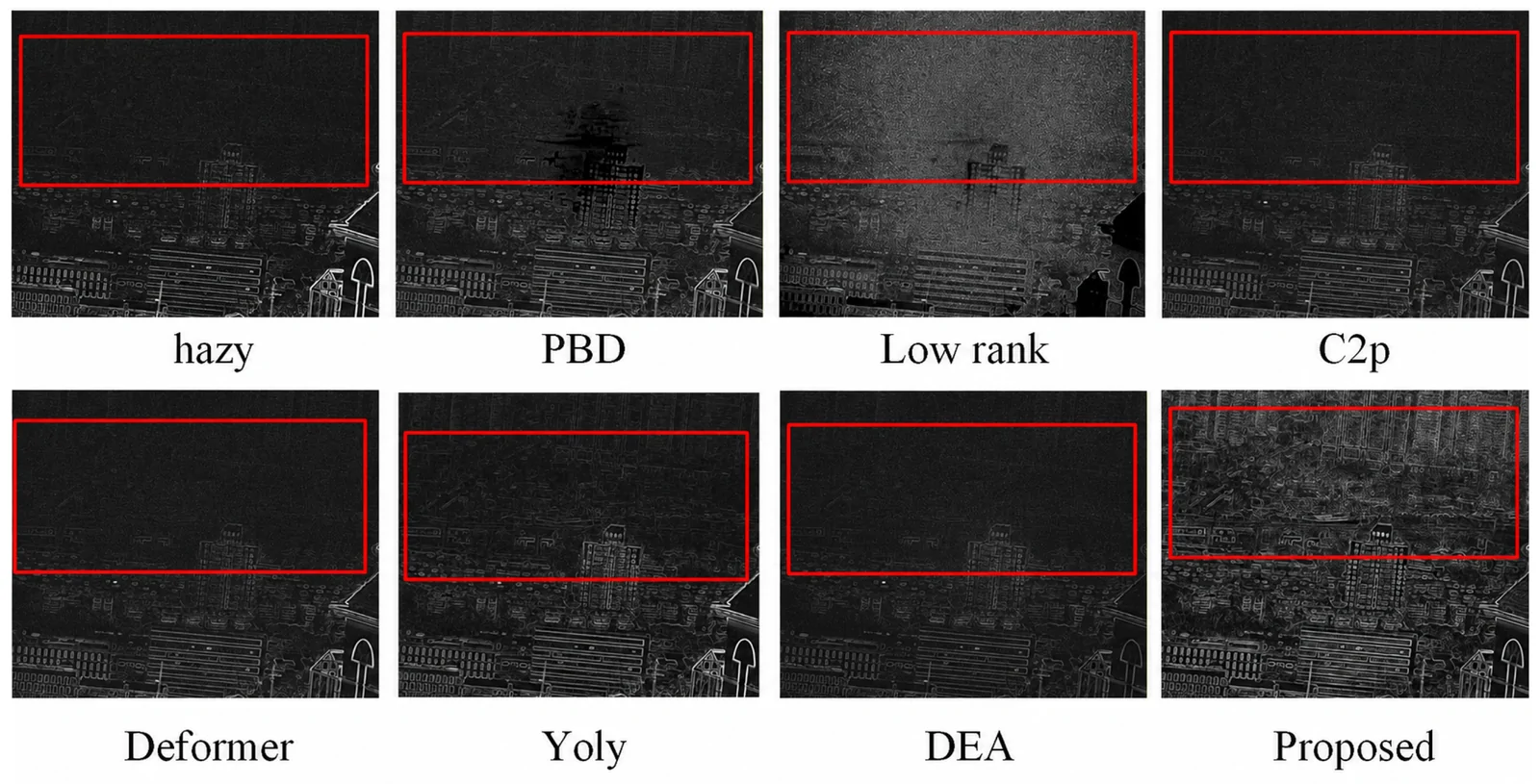
Prewitt edge maps of the dehazing results produced by different methods. The red boxes indicate the regions of interest used for comparing distant weak-edge structures.

As shown in Figure 8, the contours of distant buildings in the original hazy image are weak, and valid edges are almost undetectable in some regions. Although PBD improves visibility to a certain extent, the edges of distant buildings and fine structures remain discontinuous. Some deep learning-based methods can detect partial target contours, but they tend to produce broken edges, false edges, or missing local structures in distant regions. In contrast, the edge map obtained by the proposed method shows better continuity in building contours, tower crane structures, and distant weak-edge regions, indicating that it can recover more structural information from severely degraded hazy images. This result is consistent with the preceding visual comparisons and indicates improved preservation of weak structural contours in dense-haze scenes.

### 3.3. Quantitative Experiments

Since strictly paired haze-free ground-truth images are difficult to obtain for real hazy polarization images, no-reference image quality assessment metrics were used for quantitative evaluation, including FADE, BRISQUE, Entropy, Contrast, and STD. These metrics evaluate the dehazing results from the perspectives of haze density, natural image statistics, information content, contrast, and gray-level dispersion, respectively, and provide a comprehensive reference for real-scene dehazing performance.

**Figure 9:**
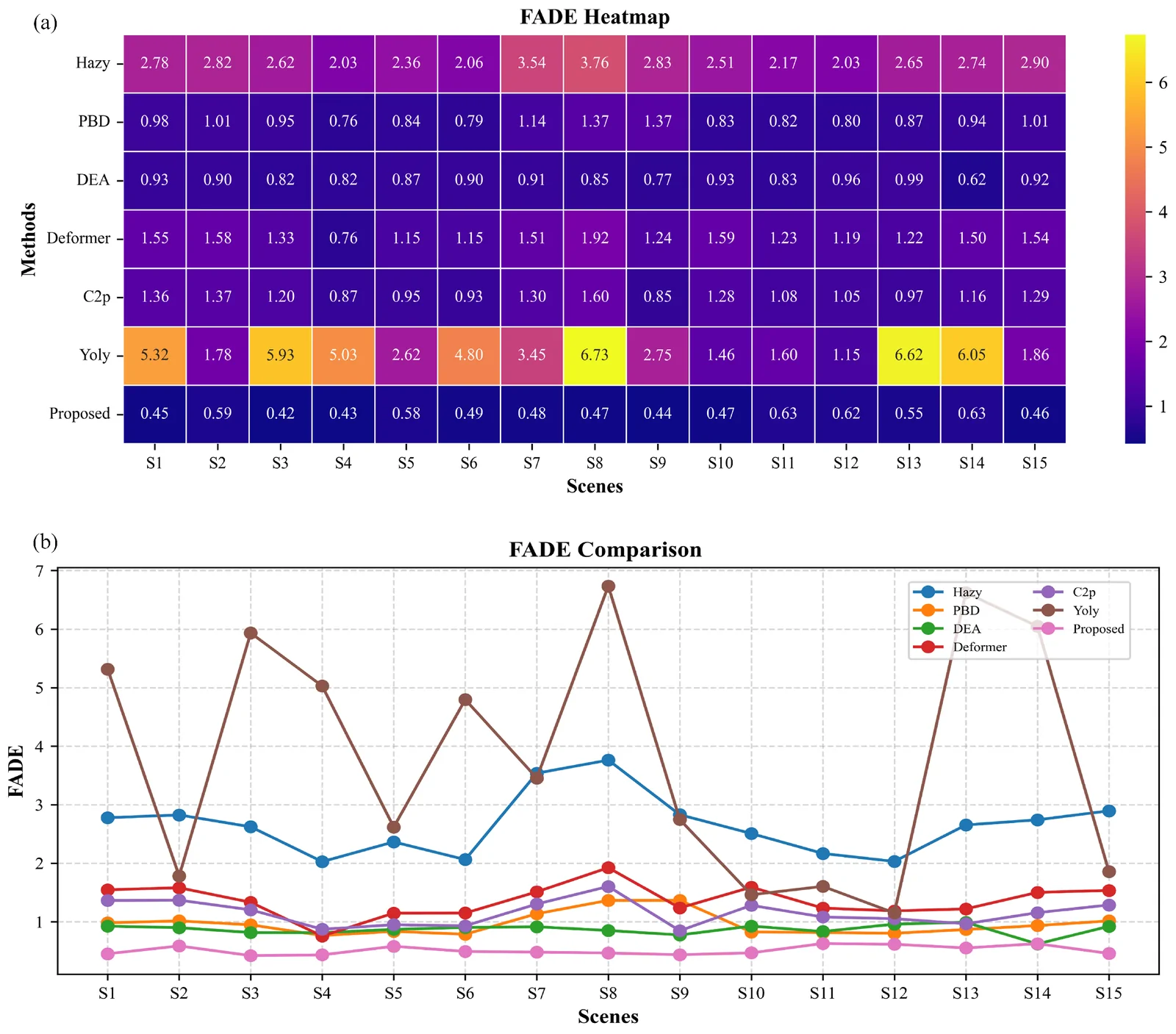
Quantitative comparison of FADE scores. (**a**) Heat map. (**b**) Line plot.

FADE is a no-reference metric based on natural image statistics and is used to estimate the haze density of an image. A lower FADE score generally indicates less haze degradation. As shown in Figure 9, the proposed method achieves the lowest FADE score, indicating its superior capability in suppressing haze density. This performance can be attributed to the AoP frequency-domain prior and the two-stage training strategy. Nevertheless, slight residual haze and noise enhancement still appear in some strongly scattering regions, suggesting that detail restoration under extremely dense haze remains challenging.

**Figure 10:**
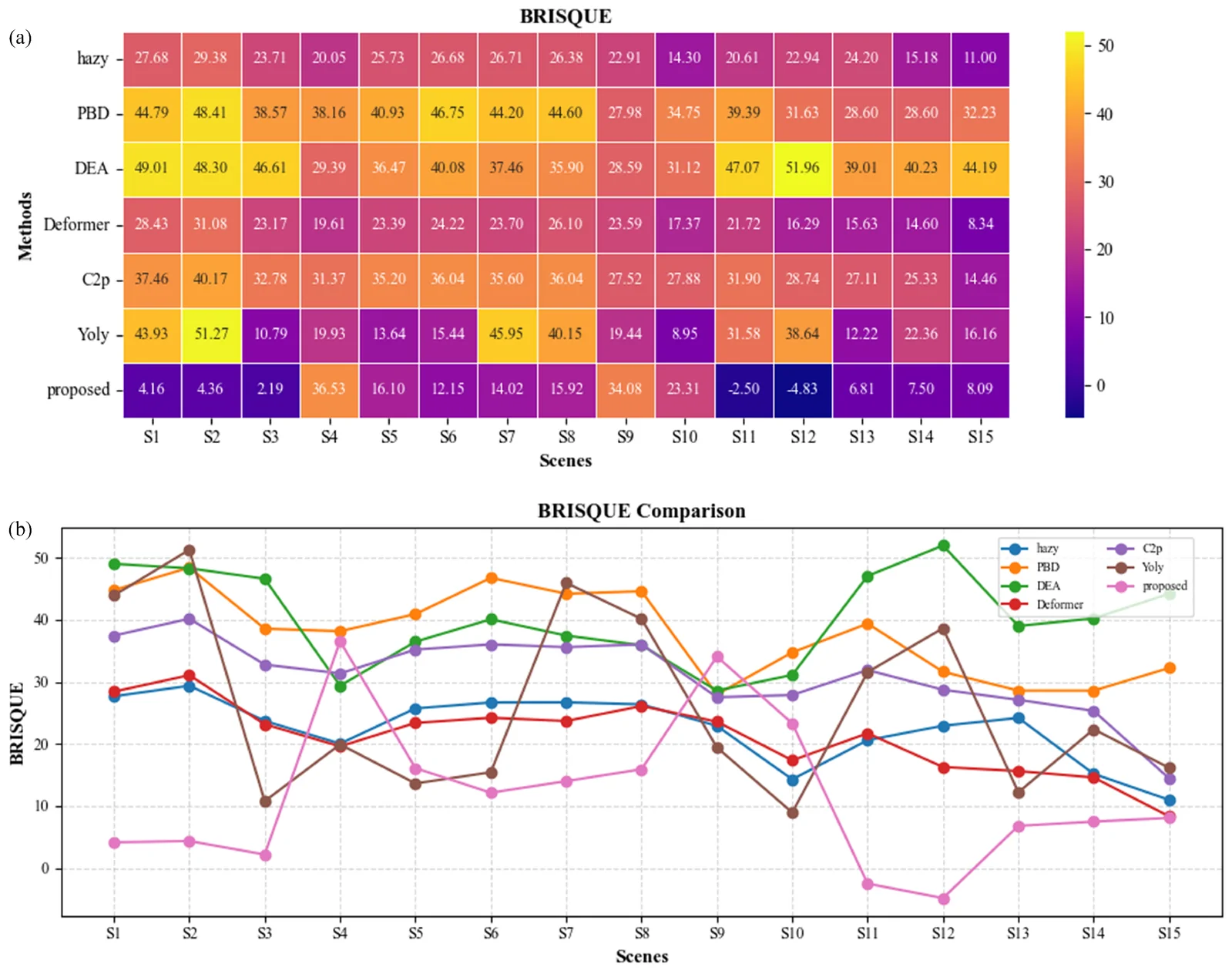
Quantitative comparison of BRISQUE scores. (**a**) Heat map. (**b**) Line plot.

Similar to FADE, BRISQUE is also a no-reference metric based on natural image statistics and is used to evaluate image naturalness and distortion. A lower BRISQUE score indicates better image quality. As shown in Figure 10, DEA-Net and C2PNet obtain relatively high BRISQUE scores. This reflects a typical issue in image quality evaluation: these methods tend to enhance image details and contrast during restoration, but such enhancement may introduce slight artifacts, such as ringing effects or noise amplification, which are penalized by BRISQUE. The proposed method achieves the lowest BRISQUE score, demonstrating that the two-stage training strategy and CLIP-based semantic regularization help improve the naturalness of the restored images.

**Figure 11:**
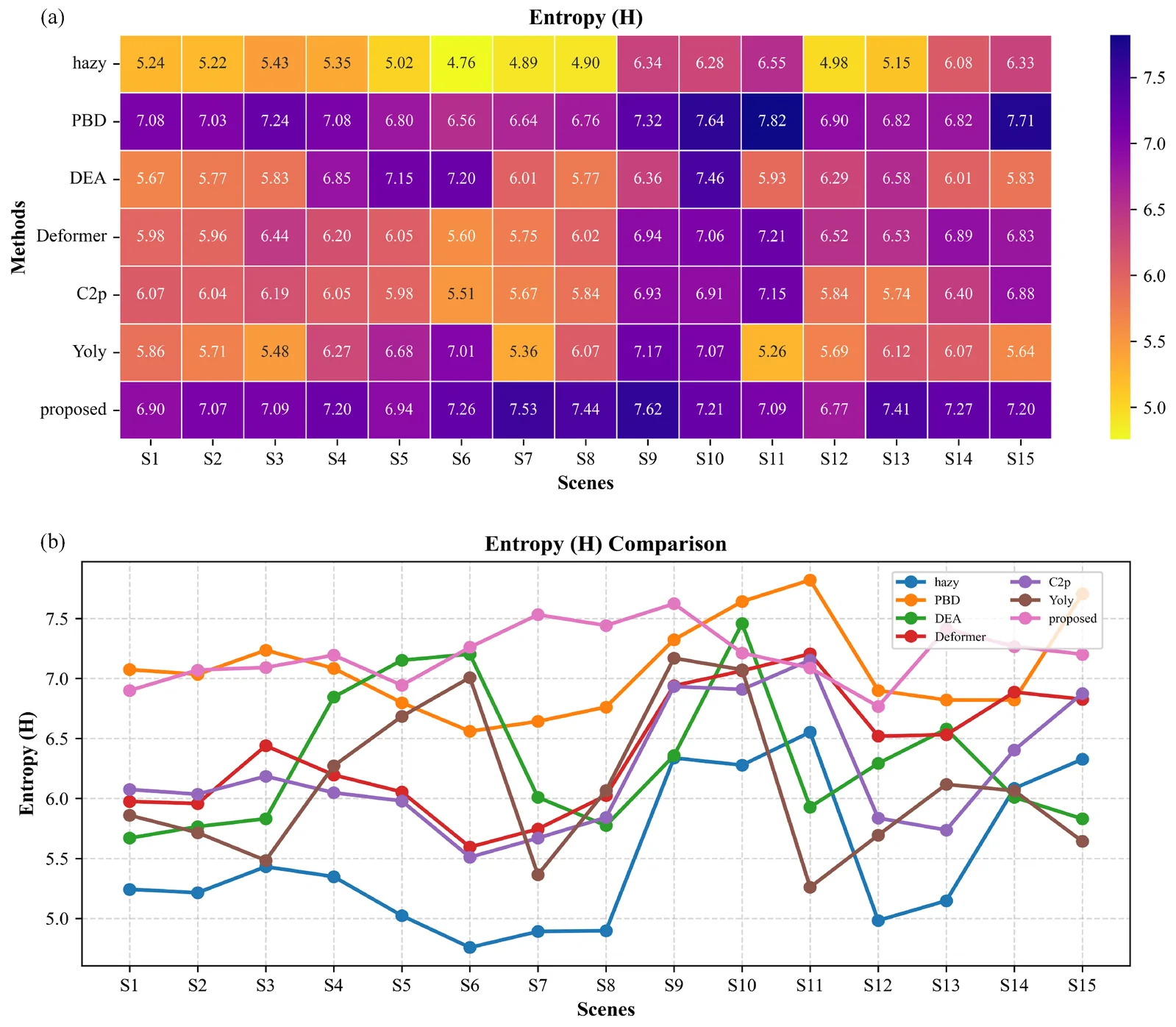
Quantitative comparison of entropy scores. (**a**) Heat map. (**b**) Line plot.

Entropy is an important metric for measuring image detail richness and information content. As shown in the line plot of Figure 11, the proposed method and PBD consistently maintain relatively high entropy values, indicating that both methods are effective in recovering information obscured or lost due to haze. Among all methods, the proposed method achieves the highest average entropy, suggesting that the two-stage training strategy can effectively recover most image details while incorporating polarization information. PBD achieves the second-highest entropy, further illustrating the benefit of incorporating atmospheric scattering physics into image restoration.

**Figure 12:**
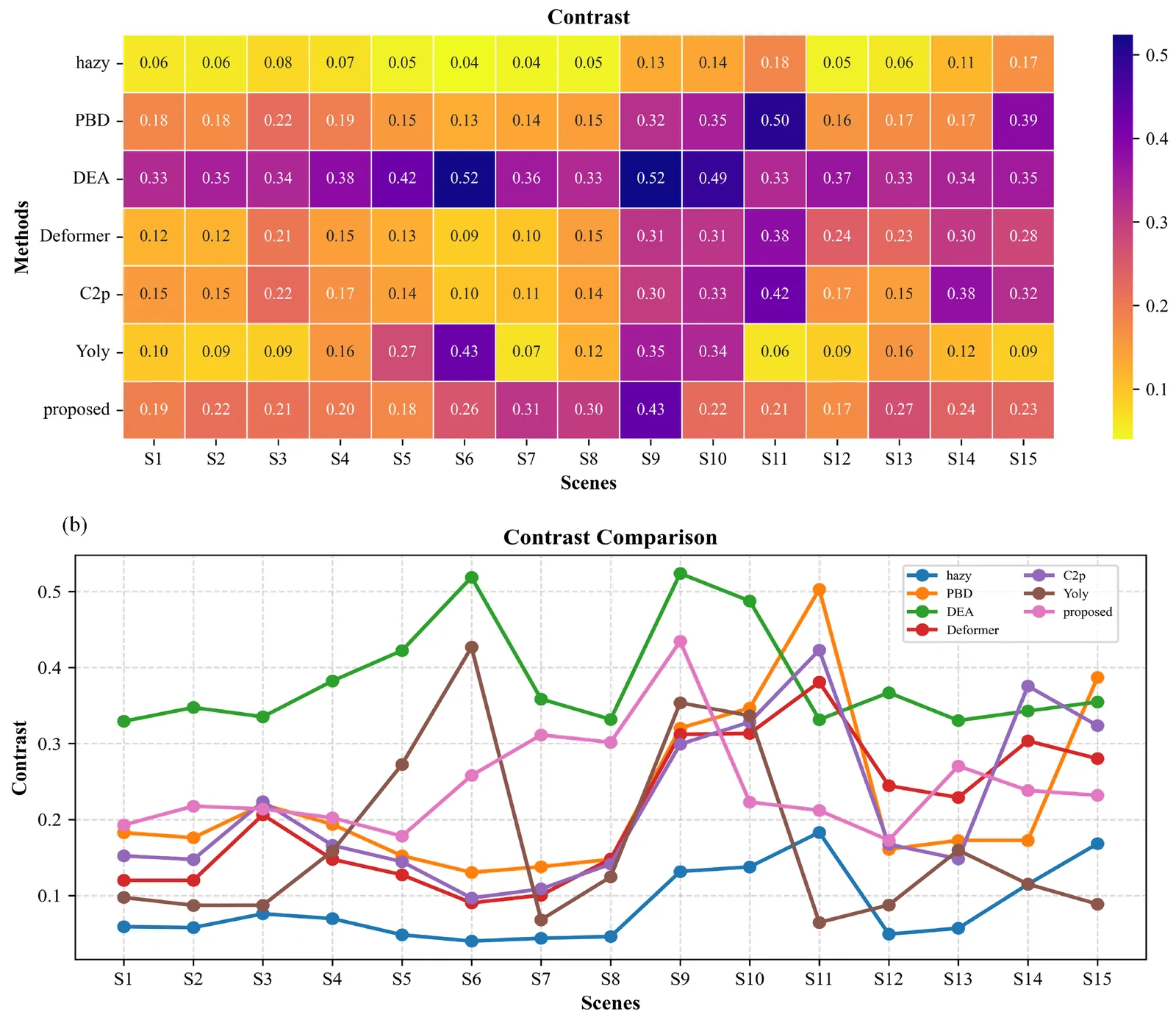
Quantitative comparison of contrast scores. (**a**) Heat map. (**b**) Line plot.

Contrast reflects the contrast level of an image, and a higher contrast is generally beneficial for improving target visibility. As shown in Figure 12, DEA-Net obtains the highest contrast value, indicating its strong contrast enhancement capability. The contrast score of the proposed method is lower than that of DEA-Net, but it remains higher than those of the original hazy images and most comparison methods. This suggests that the proposed method improves image contrast while avoiding excessive enhancement. Combined with the visual results in Figure 5 and Figure 6, it can be observed that excessively high contrast does not necessarily correspond to the most natural dehazing result, since some methods may enhance local contrast at the cost of brightness shifts or local distortions.

**Figure 13:**
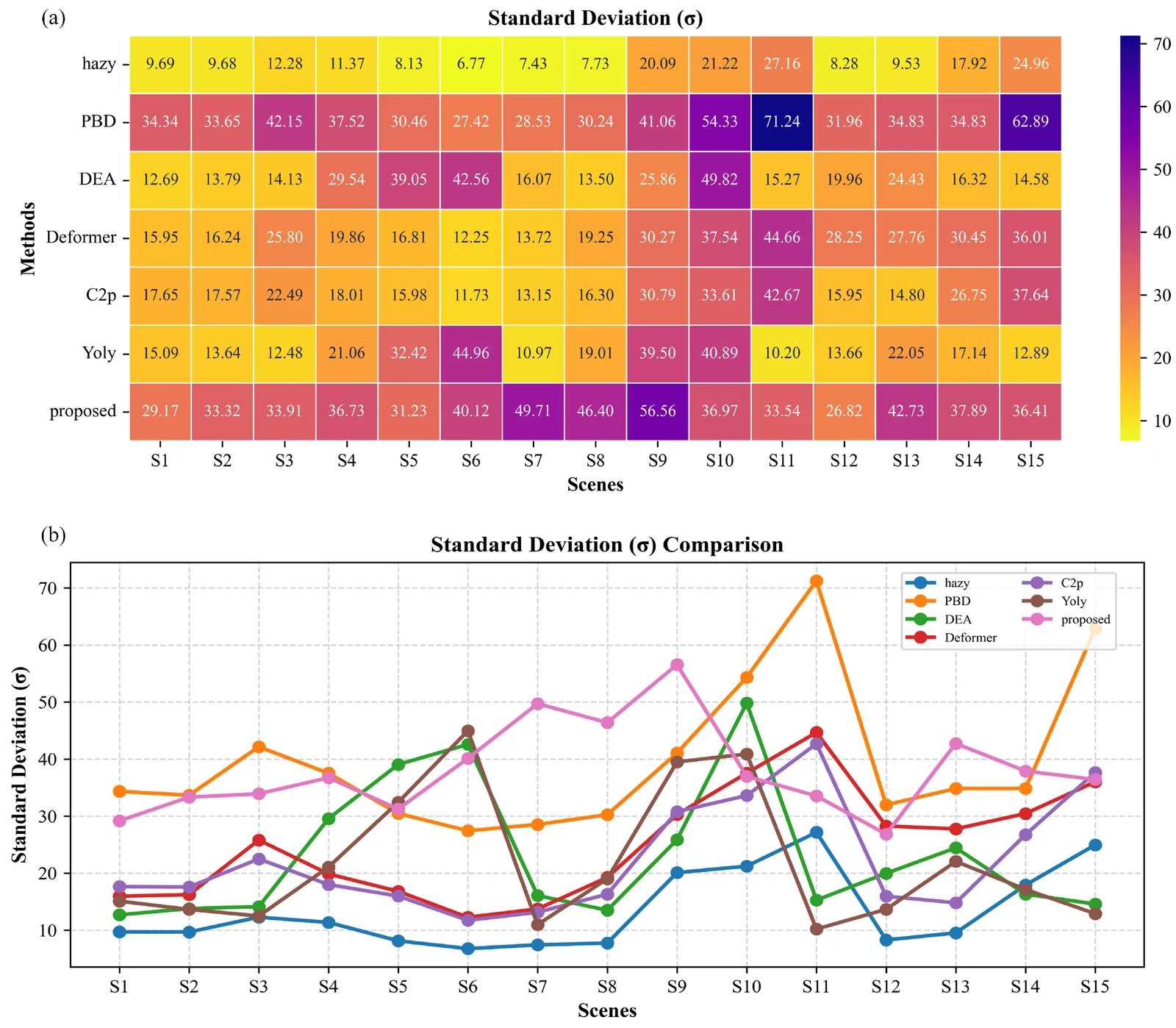
Quantitative comparison of STD scores. (**a**) Heat map. (**b**) Line plot.

A higher STD value generally indicates richer gray-level variation and stronger image contrast. As shown in Figure 13, the proposed method obtains a relatively high STD value, indicating enhanced gray-level variation. However, STD should be interpreted together with perceptual and structural metrics, since noise amplification or excessive enhancement may also increase this metric. The average scores of different methods are listed in Table 1, where the best results are highlighted in bold. Overall, the proposed method achieves the best performance on FADE, BRISQUE, and entropy and maintains competitive results on contrast and STD. These results indicate that the proposed method achieves a favorable balance among haze suppression, naturalness preservation, and information recovery. Compared with deep learning-based methods, the proposed method improves target visibility while maintaining more natural visual quality. Compared with traditional polarization-based physical methods, it further improves local structure recovery and overall image quality.

Although the proposed method achieves effective restoration in most real-world dense-haze scenes, its performance may degrade under several challenging conditions. When the atmospheric scattering is extremely strong and the object radiance is almost completely attenuated, insufficient structural information remains in the observations, leading to incomplete detail recovery. In addition, AoP estimation may become unstable in weakly polarized or low-signal regions, which can cause local intensity fluctuations or structural artifacts. The low-frequency-dominant atmospheric AoP prior may also become less effective under highly nonuniform illumination or complex polarization reflections. These limitations indicate that the proposed method relies on the presence of sufficient polarization and structural cues in the observed hazy images.

### 3.4. Ablation Study

**Figure 14:**
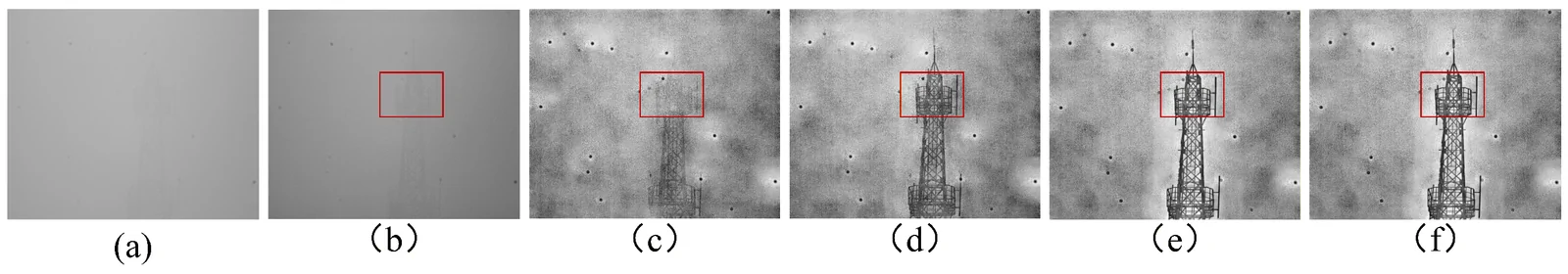
Qualitative ablation results of the proposed framework. From left to right: (**a**) hazy input, (**b**) baseline, (**c**) + AoP prior, (**d**) + two-stage optimization, (**e**) + SFDE, and (**f**) full model with CLIP semantic regularization. The red boxes indicate regions of interest for local comparison.

To evaluate the contribution of each component, an incremental ablation study was conducted under the same training and testing settings. The baseline adopts the three-branch self-supervised framework without the AoP prior, two-stage optimization, SFDE module, or CLIP semantic regularization. These components were then introduced sequentially. The qualitative and quantitative results are shown in Figure 14 and Table 2, respectively.

Compared with the baseline, introducing the AoP prior alone provides a modest improvement in FADE and entropy. This is reasonable because, under single-stage joint optimization, the AoP prior serves only as an auxiliary structural constraint and cannot independently resolve the coupling among the object-radiance field, atmospheric polarization, and transmission. After introducing the two-stage optimization strategy, the prior-guided initialization is established before joint reconstruction optimization, allowing the physical and AoP priors to be more effectively utilized and resulting in a substantial overall improvement. Incorporating SFDE further improves haze suppression and target–background separability. Finally, the full model achieves the best FADE and BRISQUE scores while maintaining competitive entropy and CNR, demonstrating the complementary effects of the proposed components.

### 3.5. Hyperparameter Sensitivity Analysis

In Stage 1, $L_{AoP}$ and $L_{prior}$ supervise different subnetworks. Specifically, $L_{AoP}$ updates only Net-J, whereas $L_{prior}$ supervises Net-A and Net-t, with no AoP gradient propagated to the atmospheric or transmission branches. Therefore, the two losses do not directly balance competing objectives on shared network parameters, and both $\lambda_{AoP}$ and $\lambda_{prior}$ are fixed to 1. The effectiveness of the AoP constraint was evaluated separately through the ablation study. In Stage 2, $L_{rec}$ and $L_{clip}$ jointly constrain Net-J. Therefore, the sensitivity of $\lambda_{clip}$ was evaluated using {0,0.25,0.5,1} while fixing $\lambda_{rec}=1$. As shown in Table 3, $\lambda_{clip}=0.5$ provides the most balanced performance across haze suppression, perceptual quality, information preservation, and target visibility, and was therefore adopted in the final model. As shown in Table 4, the proposed method incurs relatively high computational and memory costs due to the spatial-frequency feature processing. Therefore, the current implementation is not intended for real-time deployment but is more suitable for high-quality restoration of polarization images under dense-haze and strong scattering conditions.

## 4. Conclusions

This work presents a self-supervised polarization dehazing framework for real-world dense-haze conditions. By introducing the frequency-domain characteristics of atmospheric AoP as an additional physical constraint, the proposed method mitigates the ambiguity in decomposing the object-radiance polarization field, atmospheric polarization, and transmission. The two-stage optimization strategy improves the stability of physical-component estimation, while the SFDE module enhances global haze removal and local structure restoration. Experiments on real-world polarization images demonstrate favorable performance in haze suppression, information preservation, and target visibility. The ablation results further confirm that the proposed components provide complementary contributions, with the two-stage strategy producing the most substantial overall improvement.

Several limitations remain. When atmospheric scattering is extremely strong, the object-radiance information contained in the observations may be insufficient for complete structural recovery. In addition, highly nonuniform illumination and complex polarization reflections may deviate from the assumed frequency characteristics of atmospheric polarization and reduce the effectiveness of the proposed prior. The current spatial-frequency processing also introduces relatively high computational and memory costs. Future work will focus on adaptive prior modeling for more complex atmospheric conditions, lightweight network design, and validation on larger and more diverse real-world polarization datasets and downstream vision tasks.

## Figures and Tables

**Table 1:** Quantitative comparison of different dehazing methods on real-world polarization hazy images. Results are reported as mean ± standard deviation. ↑ and ↓ indicate higher-is-better and lower-is-better metrics, respectively. The best result for each metric is highlighted in bold.

Method	FADE ↓	BRISQUE ↓	Entropy ↑	Contrast ↑	STD ↑	CNR ↑
Hazy	2.6533±0.5087	22.4975±5.3592	5.5011±0.6280	0.0855±0.0484	13.4822±6.8804	1.5531±0.3235
PBD	0.9642±0.1932	37.9722±6.9982	7.0821±0.3920	0.2269±0.1102	39.6971±13.0437	1.6239±0.3151
C2P	1.1508±0.2182	31.1741±6.3636	6.2132±0.5205	0.2164±0.1049	22.3397±9.6384	1.5816±0.3408
DeFormer	1.3633±0.2756	21.1499±5.8873	6.3978±0.5063	0.2082±0.0936	24.9871±9.6951	1.6427±0.3586
YOLY	3.8100±2.0484	26.0311±14.4303	6.0985±0.6267	0.1685±0.1187	21.7305±11.8106	2.3796±0.2896
DEA	0.8680±0.0906	40.3592±7.3719	6.3139±0.5956	0.3843±0.0700	23.1710±11.9669	1.4681±0.2253
PSD	2.5892±0.7826	39.9162±2.2686	6.2249±0.3758	0.2398±0.1024	38.6707±10.2631	3.4117±0.5697
Proposed	0.5137±0.0758	11.8592±12.0276	7.2010±0.2371	0.2439±0.0666	38.1000±7.9785	2.9074±0.5526

**Table 2:** Ablation study of the proposed method on real-world polarization hazy images. ↑ and ↓ indicate higher-is-better and lower-is-better metrics, respectively. A check mark (✓) indicates that the corresponding component is included in the model configuration, and the best result for each metric is highlighted in bold. Results are reported as mean ± standard deviation.

Variant	AoP	Two-Stage	SFDE	CLIP	FADE ↓	BRISQUE ↓	Entropy ↑	CNR ↑
Hazy	–	–	–	–	2.6533±0.5087	22.4975±5.3592	5.5011±0.6280	1.5531±0.3235
Baseline	–	–	–	–	1.4791±0.1573	22.4376±6.0885	6.2602±0.4970	1.6558±0.3540
+AoP	✓	–	–	–	1.4490±0.3800	25.9167±6.6636	6.6187±0.3524	1.6414±0.3668
+Two-stage	✓	✓	–	–	0.7858±0.1299	12.5591±11.3475	7.3482±0.1044	2.9017±0.5107
+SFDE	✓	✓	✓	–	0.5261±0.1055	13.3863±10.4849	7.2419±0.2010	2.9773±0.4915
Full model	✓	✓	✓	✓	0.5137±0.0758	11.8592±12.0276	7.2010±0.2371	2.9074±0.5526

**Table 3:** Sensitivity analysis of $\lambda_{clip}$ on real-world polarization hazy images. ↑ and ↓ indicate higher-is-better and lower-is-better metrics, respectively, and the best result for each metric is highlighted in bold.

$\lambda_{\mathrm{clip}}$	FADE ↓	BRISQUE ↓	Entropy ↑	CNR ↑
0	0.5261±0.1055	13.3863±10.4849	7.2419±0.2010	2.9773±0.4915
0.25	0.5501±0.1145	9.6210±12.0836	7.1533±0.3168	2.7252±0.5217
0.5	0.5137±0.0758	11.8592±12.0276	7.2010±0.2371	2.9074±0.5526
1	0.5568±0.0838	41.2788±15.3428	7.5548±0.1935	1.4262±0.2360

**Table 4:** Computational complexity comparison of different dehazing methods. For PSD and YOLY, the reported FLOPs and processing time correspond to the complete test-time optimization process with 800 iterations. ↓ indicates that lower values are better.

Method	Params (M) ↓	FLOPs (G) ↓	Model Size (MB) ↓	Time (ms) ↓	Memory (MB) ↓
PSD	47.40	12,987.27	180.80	5303.01	256.15
C2P	67.01	4229.20	255.62	204.33	2718.58
YOLY	47.39	11,480.60	180.79	3513.34	247.98
DEA	3.65	1301.91	13.94	178.33	1526.05
DeFormer	15.93	2077.12	60.76	609.54	3307.83
Proposed	13.98	2591.74	53.33	501.65	4053.08

## Data Availability

The data presented in this study are available from the corresponding author upon reasonable request.
